# Do Spatial Boundaries Matter for Exploring the Impact of Community Green Spaces on Health?

**DOI:** 10.3390/ijerph17207529

**Published:** 2020-10-16

**Authors:** Jong Cheol Shin, Mei-Po Kwan, Diana S. Grigsby-Toussaint

**Affiliations:** 1 Department of Behavioral and Social Sciences, Center for Health Promotion and Health Equity, School of Public Health, Brown University, Providence, RI 02912, USA; 2 Department of Geography and Resource Management, The Chinese University of Hong Kong, Shatin, Hong Kong, China; mpk654@gmail.com; 3 Institute of Space and Earth Information Science, The Chinese University of Hong Kong, Shatin, Hong Kong, China; 4 Department of Human Geography and Spatial Planning, Utrecht University, 3584 CB Utrecht, The Netherlands; 5 Department of Epidemiology, School of Public Health, Brown University, Providence, RI 02912, USA

**Keywords:** green space, community activity space, environmental exposure, exercise, physical activity, health

## Abstract

Green space exposure is thought to have a positive influence on physical activity behavior and overall health. However, the literature remains equivocal, and green space measurement methods remain complicated. Using data from the Illinois Behavioral Risk Factor Surveillance System, this study examines the influence of green space on health-related factors, such as exercise, physical health, and mental health. Moreover, we explore the methods for measuring community green space via various spatial boundaries and green space resources. The results show that combining two contextually designated census boundaries and a measure of green space with seasonality were the best spatial conceptualizations for capturing community green space. Moreover, the findings showed a positive influence of green space exposure on health outcomes. These findings highlight the importance of considering geographic contexts of daily human behaviors and green space seasonality in providing a better understanding of the influence of community activity space on environmental exposure measurement. Further, this work contributes to community planning for encouraging health-promoting behaviors.

## 1. Introduction

Green space is considered one of the key environmental factors that influence human behavior and health status [1,2,3]. For example, people who live in proximity to a higher density of green space have been shown to have a lower risk of heart disease [4], circulatory disease and mortality [5], sleep [6,7], and mental health conditions [8,9,10]. Green space accessibility has also been shown to have an inverse association with cardiovascular disease [11] and stress [12], while enhancing general health [13], mental health [14], and overall well-being [2]. Several studies have examined whether more green space exposure—including exposure to a higher density of green space [15,16], better proximity and accessibility [15,17], and a higher proportion of street trees [18]—has been associated with lower obesity rates, which is a key risk factor for several major chronic diseases; however, the literature overall on green space and obesity is equivocal [19,20].

The evidence of the spatial influence of green space on physical activity and exercise remains debatable. Markevych et al. [21] claimed two types of measurements for the pathway between green space and physical activity encouragement: (1) perceptual (i.e., time spent in green space, access and attractiveness, and safety), and (2) spatial (i.e., greenness, distance, and other qualities). Several studies using spatial measurements have shown that the amount of neighborhood greenness [1,20,22] and natural environments [15] are positively associated with increased physical activity levels among children and adults. In addition to the amount of green space, accessibility to green space is also associated with increases in walking and cycling [23] and moderate-to-vigorous physical activity (MVPA) [24,25]. Chaix et al. [26] also showed that green space quality is associated with proximity for recreational walking as much as the quality of open spaces and the absence of air traffic and noise exposure. In contrast, Hillsdon et al. [27] argued that there is no evidence to support urban green spaces’ public health value on physical activity. Maas et al. [28] also indicated that the amount of green space exposure has no significant effect on sports participation and even reduces leisure-time physical activity such as walking and cycling. Moreover, Ord et al. [29] showed that individuals living in the greenest neighborhood were less likely to meet guidelines for walking.

Of note, the mediating effect of physical activity between green space and health outcomes also remains equivocal. Specifically, several studies examined the partial or full mediation effect of physical activity between urban green space and health [8], individual green space exposure and health [30], and visiting green space and mental health [31]. In contrast, some studies claimed no mediation effect of physical activity between streetscape greenery and health [32], and green space density and well-being [33].

One possible reason for the inconsistent relationship between green space, exercise, and health outcomes may be due to the lack of consistent geographic measures. One of the frame-dependency phenomena associated with geographic measurement is the modifiable areal unit problem (MAUP), which means that results may differ when different zoning schemes or spatial scales are used to obtain geographic variables [34,35]. For example, different results in built environment measurements were obtained depending on zoning (e.g., population density, grid vs. buffer) or scaling (e.g., different buffer sizes) [36,37]. Houston et al. [22] illustrated the difference between conceptualizing green space zoning using buffers and grids and the varying influences on levels of physical activity. Moreover, cumulative opportunity-based indicators, which cover all potential green space exposures, are more consistently related to health outcomes than residence-based proximity indicators [3]. Furthermore, environmental exposure is influenced by an individual’s area of mobility [30]; thus, identifying community-wide activity spaces, which are conceptualized to reflect the potential area where people live or work, should ultimately cover potential individual-level activity spaces [38]. However, there has been little to no research that considers the proper spatial boundaries of green space measurements for integration with population-level health data.

Therefore, we seek to clarify the role of green space in a community activity space and explore its effect on exercise, physical health, and mental health. We hypothesize that (1) green space will positively influence exercise and health outcomes; (2) temporality of green space measurements (i.e., the maximum greenness measurement for one year vs. the seasonal pattern of greenness measurement over the course of one year) will have different influences on the health outcomes of interest; and (3) greenspace metrics calculated based on different spatial boundary systems will have different associations with area-based measures of physical activity and health outcomes.

## 2. Methods

### 2.1. Setting

The analysis focused on the state of Illinois, the sixth largest state in the U.S. [39]. According to the American Community Survey (ACS), approximately 61.9% of Illinois’s population is non-Hispanic White, 14.1% is non-Hispanic Black, 5.2% is non-Hispanic Asian, and 16.8% is Hispanic [40]. This distribution represents a racial/ethnic distribution similar to the entire U.S. population: 61.5% non-Hispanic White, 12.3% non-Hispanic Black, 5.3% Asian, and 17.6% Hispanic [40]. Consistent with the ACS, the Illinois Department of Public Health (IDPH) classifies rural counties as counties that are not part of a metropolitan statistical area (MSA) or counties that are part of the MSA, but have a population of fewer than 60,000 people. Of the 102 counties in Illinois, 19 are classified as urban and 83 as rural.

### 2.2. Data Sources

The IDPH, a local government agency, has a repository of county-level data: the Illinois County Behavioral Risk Factor Surveys (ICBRFS), which are extensive, repeated cross-sectional health surveys that provide health information, including health behavior, sociodemographic, and disease status data. Our study included round five of the ICBRFS collected between 2010 and 2014 from Illinois adult residents aged 18 years or older [41]. Among the 102 counties, the ICBRFS covered 94 counties based on population counts that form IDPH’s administrative districts. For example, Cook County, which has the largest population in Illinois, is divided into Chicago and suburban Chicago areas, while 12 other small populated counties in Southern Illinois were reclassified to three counties: “Southern seven counties (*n* = 7)”, “Franklin-Williamson counties (*n* = 2)”, or “Egyptian Health Department (*n* = 3)”. The dataset is publicly available, but the data are provided only as a read-only web browser (JavaScript). Therefore, the following R-studio packages for web-crawling were used to acquire a readable dataset: “rvest” [42], “XML” [43], and “RCurl” [44].

Topologically Integrated Geographic Encoding and Referencing (TIGER)/line shapefiles with selected demographic and economic data derived from the 2009–2013 ACS data were used to delineate spatial boundaries for population-based residential areas and contextually designated census areas. Demographic and economic information in the ACS data, such as gender, age, race, household, and poverty status [45], was solely used for validation of the ICBRFS dataset for each county. Since the initial unit of analysis for the population-based spatial boundary measurement is the *block group*, which is the smallest geographic unit in the U.S., the numbers of households within block groups were used. The selected *block groups* aggregate into larger spatial units, such as *counties*.

Green space exposure, measured as the relative amount of vegetation in the study area, was derived from the Moderate Resolution Imaging Spectroradiometer (MODIS) from the National Aeronautics and Space Administration (NASA). The MODIS uses satellite imagery to aggregate and integrate land-cover information at the national scale, and it consists of the proportion of each 250 × 250 m area covered by tree canopy. Normalized Difference Vegetation Index (NDVI) images were downloaded from Earth Explorer [46] provided by the U.S. Geological Survey (USGS)–eMODIS Remote Sensing Phenology Products. Two types of NDVI measures of vegetation were acquired: the maximum NDVI (MXN) and the time-integrated NDVI (TIN). The MXN, which is based on the maximum of the time series of daily or monthly NDVI values during the year, can be inferred as the maximum level of vegetation in a spatial area, while the TIN represents the average greenness during the plant’s entire growing season. Both NDVI measures range in score between 0 and 100%, and higher values represent denser greenness.

### 2.3. Socio-Demographic Variables

Sociodemographic variables were used as major independent or control variables. Except for urbanicity (urban = 1, rural = 0), all variables were included as percentages of the following categories (in parentheses): age (65 years or older), gender (female), race (white), poverty (income less than $15,000), employment (employed), education (less or equal to high school degree, smoke (nonsmoker), and alcohol use. Poverty was defined as an annual household income of less than $15,000 based on the 2013 poverty guidelines for two-person households [47]. The definition of rural counties was based on the IDPH stratified county category, which indicates that the counties with more than 100,000 residences were considered as “urban”. Variables related to marital status were excluded due to large amounts of missing data and high collinearity with other covariates.

### 2.4. Health Behavior and Health Outcome Variables

As independent variables, three self-directed health behaviors (smoking, alcohol use, and exercise) and one health condition (obesity) were included in the analysis, and these variables were based on self-reported questionnaires. The exercise variable was constructed from the only physical activity-related question in the ICBRFS: “*During the past month, other than your regular job, did you participate in any physical activities or exercises such as running, calisthenics, golf, gardening, or walking for exercise?*”); this variable was used as both an independent variable and dependent variable, depending on the purpose of the analysis. The smoking variable represented a person who ever smoked, while the alcohol variable was the proportion of people at risk for chronic drinking. The prevalence of obesity was based on the number of individuals falling into specific weight categories based on self-reported body mass index (BMI), calculated based on height and weight. BMI is classified by the following criteria [48]: underweight (BMI under 18.5 kg/m^2^), normal weight (BMI between 18.5 kg/m^2^ and 25 kg/m^2^), overweight (BMI between 25 kg/m^2^ and 30 kg/m^2^), and obese (BMI over 30 kg/m^2^).

Two health outcomes—poor physical health and poor mental health, which are considered to be influenced by green space exposure—were used as the dependent variables [4,8,9,10,11,14]. All the data are in the form of the percentage of individuals diagnosed with the condition in each county. Both physical health and mental health outcomes came from the percentage of respondents who had experienced more than seven out of the past 30 days wherein their physical health (mental health) was not good [41].

### 2.5. Spatial Analysis for Community Green Space

For the community green space measurement, which is a measure of green space resources that community members are potentially exposed to, several spatial boundaries were used to identify the potential areas where community members are exposed to green space. Boundaries were estimated by integrating various configurations of smaller areas within counties. We calculated the NDVI for each spatial boundary, and then aggregated these values to counties for the analysis. Two categories of spatial boundaries were established to estimate green space exposure. First, we used a modified definition of residential density to build population-based residential areas. The original definition of residential density by the Sierra Club [49] is one household per acre as the lowest density of single-family dwellings in suburban areas. However, this definition might not be feasible for a criterion based on block groups in an urban setting; as approximately 1/5 of the counties in Illinois (*n* = 18) do not have at least one *block group* that satisfied the definition. Therefore, this definition was not suitable for county-level data analysis. Thus, our study included two measures of modified residential density, which capture the possible population-based residential block groups: (1) *at least one household per 1 hectare (1 H/1 HA)* and (2) at least *one household per 10 hectares (1 H/10 HAs)*. These criteria were established to capture even rural residential areas and they were derived from the urban area definition (i.e., 1000 people per 1 square mile) and population density of unincorporated place (34.6 people per 1 square mile).

Second, we also used three contextually designated census units to identify community activity space. The first geographic unit was *census place*, which is defined as a concentrated area of population that is organized independently, named, and locally recognized [50]. The second unit was the *urban area*, which is designed by the U.S. Census Bureau with consisting of four land-use type: (1) census delineated urbanized areas, (2) urban clusters, (3) residential population density measured at the *census block* level, and (4) patterns of nonresidential development as outlined in the urban area [51]. Last, we also included a *combination of place and urban area* unit from all the identified characteristics of subset boundaries. From these definitions, a *census place* can conceivably encompass practical areas where people live or work, and this is closely connected to daily human activity and an individual’s potential exposure to the environment.

In addition to the three context-based boundaries and two population-based boundaries, twelve green space values (i.e., a combination of 6 spatial boundaries—*county, 1H/1HA, 1H/10HAs, census place, urban area, and combination of place and urban area*—and two types of NDVI measurements—MXN and TIN) were extracted. Comparisons within the six spatial boundaries were used to examine the zoning effect. Additionally, a subset or complement set for each spatial boundary can be used to ascertain the contextual and characteristic measurements and the differences in the effects related to exercise. Last, for the *combination of place and urban area* unit, four types of buffers (i.e., no buffer, 300 m, 500 m, and 1000 m) were established to examine the scaling effect. All of the geoprocessing and spatial analyses were performed using ArcGIS 10.7 (Environmental Systems Research Institute, Redlands, CA, USA) [52].

### 2.6. Statistical Analysis

RStudio 1.2.1335 (RStudio Team, Boston, MA, USA) [53] was used to calculate the descriptive statistics and perform t-tests and multiple regression analysis. Two-sample t-tests between urban and rural areas were performed to examine possible disparities in the sociodemographic variables, health outcomes, and green space exposure. Levene’s test was performed to check the equality of variances, and Welch’s t-test was performed instead of the two-sample t-test when this assumption did not hold. Multiple regressions analyses were performed to compare the effect of exercise within each spatial boundary and buffers regarding contextually mismatched community green space and to examine the key factors for the health outcomes of interest. The first regression analysis was the comparison between TIN and MXN explained contextual green space measurement and its influence on exercise. In addition, the comparison within the six spatial boundaries was used to examine the zoning effect, while the comparison among the buffers (i.e., no buffer, 300, 500, and 1000 m) accounted for the scaling effect. The second regression analysis examines the association between the independent variables and health outcomes with certain spatial boundaries based on the best result from the spatial contextual analysis.

## 3. Results

### 3.1. Descriptive Statistics: Study Area and Data Feasibility

**Figure 1:**
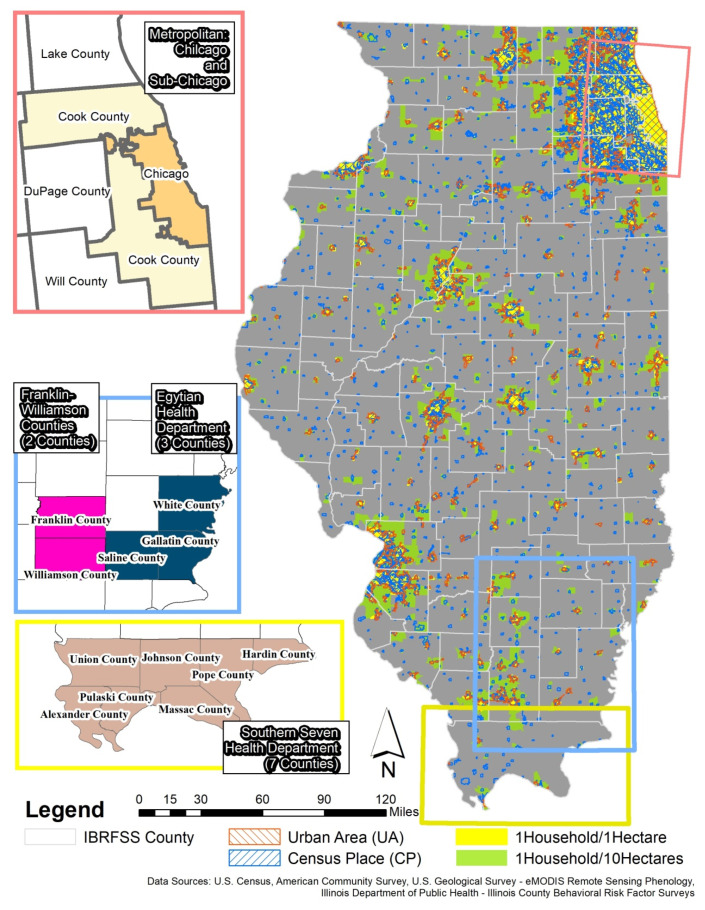
Selected counties in Illinois County Behavioral Risk Factor Surveys (ICBRFS) and spatial boundaries to identify community green space in the study area.

A total of 94 counties (20 urban and 74 rural counties) are included in the final analysis. Figure 1 illustrates the list of counties in the final analysis and each spatial boundary used to measure community green space. Seven counties (i.e., Calhoun, Cumberland, Edward, Marshall, Putnam, Scott, and Stark County) out of the 94 counties did not have any *urban area*, and one county did not meet the criteria for *1 H/10 HAs*.

**Figure 2:**
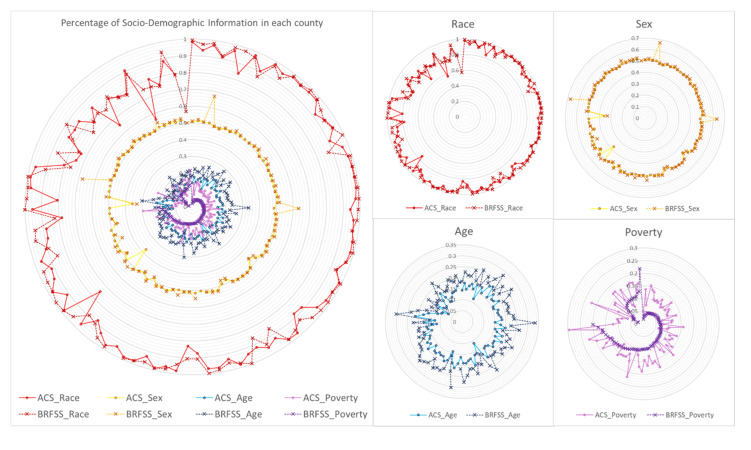
Sociodemographic comparison between Illinois County Behavioral Risk Factor Surveys and American Community Survey data.

Figure 2 illustrates the county-level demographic components by comparing the primary dataset (ICBRFS) with the reference dataset (ACS), which estimates the total population. For race and gender, the percentages of white and female in each county and its pattern show the similarity between ICBRFS and ACS. The percentage of the elderly is higher than that in the ACS data, but both had similar patterns. Lastly, the percentage and pattern of poverty are significantly different between ICBRFS and ACS. One of the reasons for the difference is the different criteria regarding poverty between ICBRFS and ACS. Specifically, ICBRFS only provided household income without household size, unlike the ACS where poverty is calculated by both household size and household income. With this limitation, the poverty criterion for ICBRFS was estimated by the cut-off was less than $15,000, which covers a minimum number of household sizes to avoid type 1 error.

Table 1 presents the summary statistics for all variables regarding sociodemographic, health, and green space variables. Based on the normality test result, two-sample t-test or Welch’s t-test were performed to compare the urban and rural counties. For the sociodemographic factors, White (t = −8.853, *p* < 0.001) and elderly populations (t = −6.652, *p* < 0.001) are more concentrated in rural counties. Urban counties had a higher proportion of high school graduates (t = 6.723, *p* < 0.001); this finding indicates that urban counties had a more highly educated population. There are no significant differences in the percentages of females, working population, or poverty level between urban and rural counties. For the health behaviors, larger proportions of the exercise population (t = 2.194, *p* = 0.031) and nonsmoker group (t = −2.351, *p* = 0.021) were found in urban areas. Furthermore, rural counties had more people at risk of chronic drinking than urban counties (t = −3.171, *p* = 0.002). There was no significant difference between urban and rural counties in terms of obesity. Both physical health problems (t = −2.156, *p* = 0.034) and mental health problems (t = 3.921, *p*< 0.001) occurs more often in urban counties.

**Figure 3:**
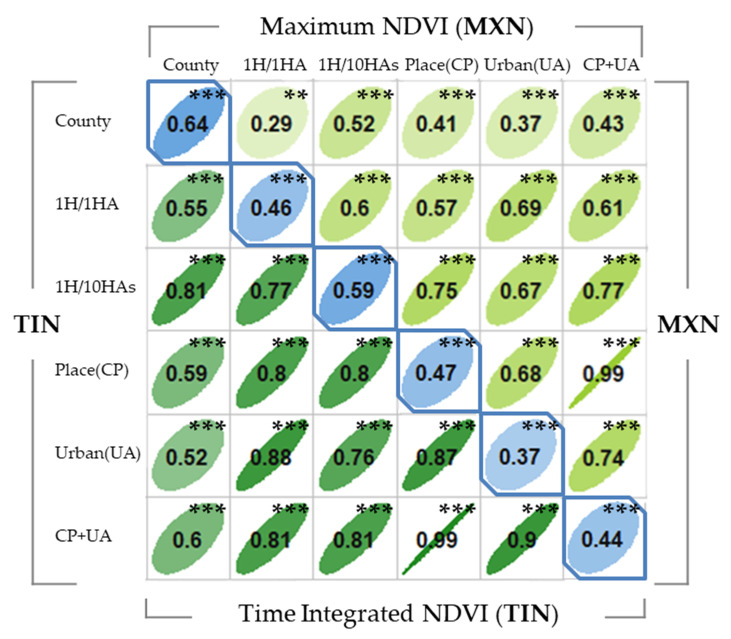
Correlation of each spatial boundary of maximum NDVI and time-integrated NDVI in Illinois.

The correlation plot in Figure 3 illustrates the relationship between the types of green space measurements used in this study. The correlation between TIN and MXN mostly showed moderate correlation (0.4~0.6), but a high correlation at the *county level* (r = 0.64) and low correlation in *urban areas* (r = 0.37). *County*-level measurements also have a relatively lower correlation to other spatial units, while *census place* and *combined* boundary showed the highest correlation between MXN and TIN (r = 0.99). Regarding the comparison among different spatial units, TIN had moderate to perfect correlation (0.52~0.9), while MXN showed low to high correlation (0.29~0.77)).

### 3.2. Spatial Boundary and Geographic Context for Community Green Space

**Figure 4:**
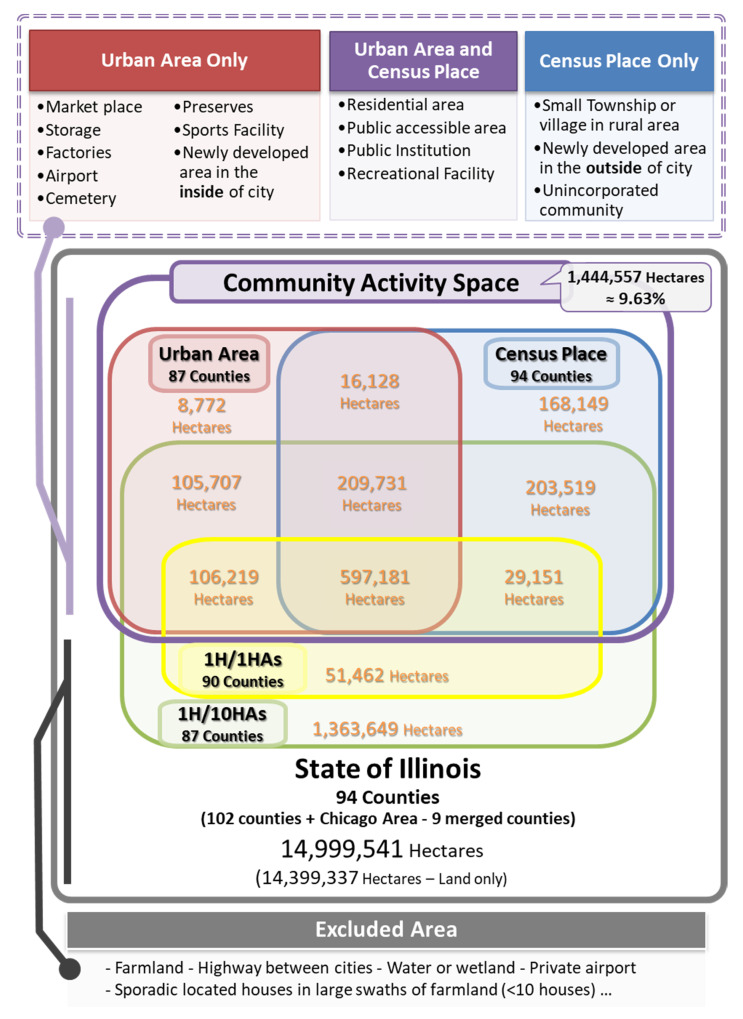
Geographical and contextual information among various spatial boundaries to determine community activity space for community green space measurement in Illinois.

Figure 4 illustrates the area and proportion of each spatial boundary and the contextual components for the mismatched places regarding community activity space classification. As seen in Figure 1, unincorporated communities and small villages are found in both the *place* and *1 H/10 HAs* units but are rarely found in *urban areas* except near urban counties. All *1 H/1 HA* areas are a subset of the *1 H/10 HAs areas*. From the diagram, 19.86% of Illinois is a part of the study area with at least one spatial boundary; other *non-boundary areas* (80.14% of Illinois) are excluded. Including parts of *1 H/1 HA and 1 H/10 HAs* (9.82% of Illinois), the following agricultural or non-daily activity related area are also excluded: farmland, highway, water or wetland, and private airport. In contrast, parts of the *urban areas* (0.06% of Illinois) or *census places* (1.16% of Illinois) consist mainly of three land-use types: commercial facilities, human activity-related natural resources, and residential areas. These areas are related to potential human activity. Based on each spatial boundary’s component and characteristics, *the combination of census places and urban areas* (9.63% of Illinois) could be considered community activity spaces.

Table 2 and Table 3 show the spatial influences of green space measurement methods and various spatial boundaries that considered the zoning and scaling effects. From Table 2, the TIN had a positive association with exercise in all of the spatial boundaries and models, while MXN had no significant association with exercise.

Table 3 illustrates the influence of the different buffer sizes concerning the *combination of census places and urban areas*. Regardless of the buffer size, the TIN was consistently significantly associated only with exercise (*p* < 0.01).

### 3.3. Regression Analysis: Risk Factors for Health Behavior and Health Outcomes

Multiple regression analyses were performed to examine the association between green space, exercise, poor physical health, and poor mental health. Table 4 represents the multiple regression models, either adjusted or unadjusted, for thirteen predictors. As seen in Table 3, the TIN showed a positive association with exercise (B: unstandardized beta = 0.803, SE: standard error = 0.277, *p* = 0.005). Although green space was negatively associated with both poor physical health and mental health, it was not statistically significant. After adjustment for control variables, heart disease and mental health show one or two significant risk factors. Counties with more females reported to have more individuals with physical health problems (B = 0.621, SE = 0.188, *p* = 0.001) and mental health problems (B = 0.749, SE = 0.183, *p* < 0.001). Moreover, more people have physical health issues (B = 0.377, SE = 0.172, *p* = 0.031) and mental health issues (B = 0.340, SE = 0.168, *p* = 0.046) in the counties with a higher prevalence of poverty. Lastly, mental health problems are less likely to occur the counties with the elderly population (B = −0.917, SE = 0.200, *p* < 0.001).

## 4. Discussion

### 4.1. Empirical Approaches of Spatial Boundaries for Community Green Space

Spatial and temporal frame dependence influences environmental exposure measurements for chronic health conditions [54]. As such, the selection of geographic units in studies exploring environmental influences on health should be nuanced. This study illustrates the spatial influences of different measurement units and conceptualizations of community green space to address the modifiable areal unit problem (MAUP) [34] and the uncertain geographic context problem (UGCoP) [54].

The MAUP may be caused by zoning and scaling effects due to unsuitable shapes and geographic units being used as the units of analysis [34]. Using large administrative units without considering the MAUP and any additional geoprocessing step is not appropriate for capturing environmental exposure and human activity coverage [55], so it needs to be tailored to the populations of interest. For example, Coutts et al. [24] examined green space at the whole-county level for the U.S. and could not capture places related to physical activity. Identifying and adopting the best areal division, zoning scheme, and spatial scale are the most popular methods to address the MAUP [54]. According to Kwan [54], the smallest areal unit of data should be used as often as possible, as it is not limited to arbitrary spatial-temporal units when individual-level space-time data are not available. Therefore, our study aggregated the *census block group*, which is the smallest geographical unit publicly available in the U.S., to mitigate the zoning issue.

Environmental exposures should also consider human behavior and their contexts, such as people’s space-time trajectories [38]. In this respect, population-based residential areas may not adequately capture the actual area of people’s daytime environmental exposure. According to Kwan, an individual’s exposure derived with his/her residence-based neighborhood may under- or overestimate the actual exposure due to the neglect of the persons’ daily mobility such as and commuting and the geographic segregation within the community [38]. Therefore, spatial environmental data need to account for adequate geographic contexts, such as an individual’s daily mobility area or potential activity area. In addition to the zoning issues due to the use of slightly larger geographic units (i.e., *postcodes* and *census area statistics wards*) than *block group*, home-based measurement [27,29] may not cover all exposure to daytime green space, such as during commuting. In this sense, community activity spaces may provide more accurate results of daily environmental exposure by representing the potential activity area and accessibility of resources within the community.

In contrast to the designated boundaries of geographic context, the population-based residential boundaries had difficulty identifying the community activity spaces. Although most of the *1 H/1 HA* boundaries are part of the core and central urban areas (93.5%), they cover only half of the community activity spaces (50.7%). Moreover, most of the 1 H/10HAs boundaries (46.9%) consist of non-human-related areas. Population-based spatial boundaries using administrative units were inaccurate, especially in rural areas; this limitation of the adoption of geographic units may be caused by the heterogeneity in the size of the *census block groups* and large target areas with spatial variability. In a study by Johnson et al. [56], *block groups* were too large to capture daily environmental exposures in rural areas but too small to do the same in urban areas.

Meanwhile, contextually designated census boundaries cover very different land-use components. For example, *urban areas* are more accurately covered than commercial facilities, inner-city natural resources, and city boundaries, but there is much missing relevant spatial data in rural areas. In contrast, *census places* can capture small townships and cities in rural areas, but less accurately capture the city’s commercial facilities and natural resources. Therefore, *the combination of census places and urban areas* will be the best option for the nationwide measurement of community activity space.

National-level surveys usually provide larger geographic units (e.g., county or state) without an individual’s identifiable information due to confidentiality concerns. In terms of scaling, using buffers for each boundary may not be appropriate for survey data. Buffer is widely used to delineate target areas’ proximity, which shows potential exposure near residential areas. Buffers are also used to weight the actual accessible green space considering complex routing and accessibility problems from spatial heterogeneity (e.g., network buffers), and they are considered one of the most applicable methods to measure the individual-level environment exposures [22,30]. However, buffers seem to be limited to applications involving census-based spatial boundaries, unlike individual-level analysis. A study by Coutts and colleagues of Florida showed no difference among four buffer levels (e.g., *0.25*, *0.5*, *1*, and *10 miles*) based on *census tracts* with population-weighted accessibility [24]. In addition, the results illustrated in Table 3 show no influence of buffer size on green space and dimensions of health, supporting the argument that buffers are not necessary for spatial analysis in large geographical units such as counties.

### 4.2. Green Space Measurements and Their Influence on Exercise

Our results indicate that community green space exposure is positively associated with the prevalence of exercise in a community; this finding aligns with a previous study on the influence of daily greenspace exposure on individual health and physical activity [30]. Moreover, the results show that the influence of green space exposure on exercise depends on the contexts of green space measurement. As shown in Figure 3, MXN was not significantly associated with the amount of exercise engaged in by the population (*p* > 0.05), while TIN had a positive association (*p* < 0.01). One possible reason for the nonsignificant result of the MXN is the seasonal effect. The MXN is measured in the period with the most vegetation, but it also indicates that measurement is happening during the hottest season in a year; thus, high temperature may decrease the amount of exercise the population engages in. In contrast, the TIN reflects the interactions between vegetation growth and the natural environment. Since this cycle is consistent with the optimal season for outdoor activity or exercising, the TIN may be a more accurate measure than MXN for green space exposure related to human activity. This phenomenon may have been related to similar issues in the literature. Fan et al. [12] found that the NDVI, which is collected from June to July (the same period as for the MXN measurement), was not statistically associated with physical activity; while other green space measurements (i.e., park acreage and distance to the nearest park) have a positive influence on physical activity and negative influence on perceived stress.

Furthermore, data sources for green space should be used carefully. Studies using land-cover or land-use data that include agricultural areas as part of green space exposure have found a negative association with walking and other types of physical activity [28,29]. Conversely, studies exploring the association between green space and physical activity using data that excludes agricultural areas have found a positive relationship [24,57]. The discrepancy in the results may be due to the general inaccessibility of agricultural land. This may lead to the UGCoP, as typical daily human activities would not encompass land used for agricultural purposes.

### 4.3. Study Limitations

There are some limitations to this study. First, green space measurements are based on satellite images that do not fully cover all contextual factors associated with green space usage. For example, proximity to green space may not be related to green space usage due to accessibility issues. Since the perceived quality of green space influences human behavior [57], both qualitative and quantitative green space measurements are required to better understand the context of human activity. Furthermore, the 250 m × 250 m resolution of the satellite images might ignore some part of the nearest residential green space in urban areas. Specifically, satellite imagery cannot distinguish places smaller than the resolution.

The ICBRFS also has some limitations. One limitation is the sample size. Unlike the Behavioral Risk Factor Surveillance System (BRFSS) of the Centers for Disease Control and Prevention (CDC), which encompasses the entire U.S., the ICBRFS covered only Illinois, which may be influenced by unique geographic characteristics compared to other U.S. states (e.g., a large agricultural sector). Additionally, the ICBRFS provides aggregated data for each county; therefore, the sample size is smaller than the actual data collected. Moreover, marital status and property type were excluded because of the large number of missing values.

Furthermore, the questionnaire did not capture actual physical activity. For example, it is uncertain that the question “*any exercise experience in the past 30 days*” adequately identifies individuals who meet the target amount of physical activity. Additionally, the BRFSS is conducted by self-report, so it is less reliable than objective accelerometer measurements and leads to inconsistent results between self-report and objective measurement [58].

Therefore, future studies should use higher-resolution satellite images and larger sample sizes. Additionally, individual-level demographic information should be considered to reflect the uniqueness of each chronic disease. Moreover, future studies should use objective measurements of physical activity and community green space and use subjective measurements to increase the contextual validity of the objective measurements.

## 5. Conclusions

Our study highlights the importance of re-conceptualizing geographic context to capture environmental influences (e.g., green space exposure) on health behaviors and health outcomes. The varying geographic conceptualizations explored in our study will be helpful for future studies using census data.

The study provides insight into gaps, in terms of health and sociodemographic factors between urban and rural areas in Illinois, and allows us to determine a key/target factor to promote physical activity and prevent chronic diseases. With practical approaches for community-level environment measurements, we found that the amount of green space in a community was positively associated with exercise, but less so with poor physical or mental health. Our study’s findings are expected to help health practitioners and policymakers better classify community areas that impact health behaviors and health outcomes.

## Figures and Tables

**Table 1:** Descriptive statistics and comparison between urban and rural counties in reported socio-economic status variables, health-related variables, and environmental characteristics: Illinois County Behavioral Risk Factor Surveys 2010–2014. (*n* = 94).

Variables	Illinois	Urban County	Rural County	Comparison: Urban Rural	
	Mean (SE)	Mean (SE)	Mean (SE)	Mean Difference (SE Difference)	*p*-Value
Socio-demographic (%)					
Sex: Female	51.32 (4)	52.47 (3.82)	51.01 (4.02)	1.46 (1)	0.150
Age: 65+ yrs.	21.5 (3.95)	17.19 (2.82)	22.66 (3.37)	−5.47 (0.82)	0.000 ***
Race: White	92.4 (8.49)	79.53 (7.97)	95.88 (4.2)	−16.35 (1.85)	0.000 ***
Poverty: < $15K	8.84 (3.98)	8.81 (4.61)	8.84 (3.83)	−0.04 (1.01)	0.969
Employment: Employed	55.62 (6.31)	57.65 (5.63)	55.08 (6.4)	2.57 (1.58)	0.107
Education: > High school	56.6 (7.73)	65.09 (7.9)	54.31 (5.9)	10.78 (1.6)	0.000 ***
Health behavior (%)					
Smoking: Ever smoker	44.6 (6.49)	41.65 (6.2)	45.4 (6.37)	−3.76 (1.6)	0.021 *
Alcohol: Chronic drinking	6.02 (3.25)	4.67 (1.6)	6.41 (3.49)	−1.74 (0.55)	0.002 **
Obesity: Obese	31.3 (5.56)	29.4 (5.52)	31.81 (5.5)	−2.42 (1.39)	0.084
Exercise: Any exercise in 30 days	74.63 (5.31)	76.9 (6.21)	74.02 (4.9)	2.88 (1.31)	0.031 *
Health outcomes (%)					
Physical health: Not good	37.36 (5.47)	39.72 (5.04)	36.72 (5.44)	3 (1.35)	0.029 *
Mental health: Not good	35.2 (5.83)	39.27 (5.97)	34.1 (5.31)	5.17 (1.37)	0.000 ***
Green space (%)					
MXN–IL	72.16 (2.71)	70.18 (4.27)	72.7 (1.8)	−2.52 (0.98)	0.018 *
MXN–1H/1HA	67.45 (3.68)	65.83 (3)	67.91 (3.75)	−2.08 (0.91)	0.025 *
MXN–1H/10HAs	70.03 (4.04)	68.6 (3.81)	70.42 (4.04)	−1.83 (1.01)	0.073
MXN–CP	67.74 (3.75)	65.95 (2.91)	68.23 (3.81)	−2.28 (0.92)	0.015 *
MXN–UA	66.58 (3.46)	65.53 (2.89)	66.9 (3.57)	−1.36 (0.87)	0.123
MXN–CP+UA	67.85 (3.68)	66.21 (3.02)	68.3 (3.74)	−2.09 (0.91)	0.024 *
TIN–IL	18.42 (2.55)	17.75 (2.95)	18.6 (2.41)	−0.85 (0.64)	0.187
TIN–1H/1HA	14.08 (2.08)	14.26 (1.9)	14.02 (2.13)	0.24 (0.53)	0.654
TIN–1H/10HAs	16.66 (2.47)	16.52 (2.56)	16.7 (2.46)	−0.18 (0.63)	0.776
TIN–CP	14.65 (1.93)	14.59 (2.21)	14.67 (1.86)	−0.08 (0.49)	0.875
TIN–UA	13.64 (2.05)	14.2 (1.98)	13.48 (2.06)	0.72 (0.52)	0.169
TIN–CP+UA	14.74 (1.9)	14.8 (2.2)	14.73 (1.83)	0.07 (0.48)	0.881

*** *p* < 0.001, ** *p* < 0.01, * *p* < 0.05; Welch’s *t*-test due to the unsatisfied assumption of equal variance. MXN: Maximum Normalized Difference Vegetation Index (NDVI), TIN: Time Integrated NDVI, H: Household, HA: Hectare, CP: Census Place, UA: Urban Area.

**Table 2:** Associations between green space and exercise using various combinations of spatial boundaries and green space measurements in Illinois.

	Maximum NDVI (MXN)		Time-Integrated NDVI (TIN)	
Model 1 ^a^	B (SE)	R^2^ (Adj. R^2^)	B (SE)	R^2^ (Adj. R^2^)
Entire County	0.012 (0.204)	0 (−0.011)	0.486 (0.211) *	0.054 (0.044)
1 Household/1 Hectare	0.138 (0.147)	0.01 (−0.001)	0.994 (0.240) ***	0.163 (0.154)
1 Household/10 Hectares	0.256 (0.132)	0.039 (0.029)	0.723 (0.208) ***	0.118 (0.108)
Urban Area (UA)	0.292 (0.163)	0.036 (0.025)	1.075 (0.254) ***	0.174 (0.164)
Place (CP)	0.163 (0.147)	0.013 (0.002)	0.763 (0.276) **	0.077 (0.067)
Combined (UA + CP)	0.170 (0.149)	0.014 (0.003)	0.822 (0.278) **	0.087 (0.077)
**Model 2 ^b^**	**B (SE)**	**R^2^ (Adj. R^2^)**	**B (SE)**	**R^2^ (Adj. R^2^)**
Entire County	0.042 (0.242)	0.218 (0.107)	0.533 (0.256) *	0.267 (0.163)
1 Household/1 Hectare	0.186 (0.161)	0.255 (0.144)	0.877 (0.240) ***	0.358 (0.262)
1 Household/10 Hectares	0.287 (0.137) *	0.283 (0.181)	0.712 (0.205) ***	0.345 (0.251)
Urban Area (UA)	0.234 (0.176)	0.280 (0.168)	0.948 (0.252) ***	0.384 (0.289)
Place (CP)	0.207 (0.160)	0.224 (0.126)	0.763 (0.274) **	0.288 (0.188)
Combined (UA + CP)	0.200 (0.164)	0.232 (0.124)	0.803 (0.277) **	0.294 (0.194)

*** *p* < 0.001, ** *p* < 0.01, * *p* < 0.05; ^a^ Null model: Exercise ~ greenspace; ^b^ Adjusted for urban, age, sex, race, employment, poverty, education, smoking, alcohol, obesity; NDVI: Normalized Difference Vegetation Index.

**Table 3:** Multiple regression analysis for exercise in varying buffer sizes of green space boundaries in Illinois.

Dependent Variable	Exercise							
Variables	No Buffer		300 m Buffer		500 m Buffer		1000 m Buffer	
	B (SE)	β	B (SE)	β	B (SE)	β	B (SE)	β
**Green space**: *time-integrated* *NDVI—place and urban area*	0.8 (0.28) **	**0.29**	0.8 (0.27) **	**0.29**	0.81 (0.26) **	**0.31**	0.76 (0.25) **	**0.32**
**Urban**: *urban county (yes/no)*	0 (0.02)	0.03	0.01 (0.02)	0.06	0.01 (0.02)	0.06	0.01 (0.02)	0.06
**Sex**: *female*	0.16 (0.19)	0.11	0.12 (0.19)	0.08	0.11 (0.19)	0.07	0.1 (0.19)	0.07
**Age**: *elderly (65+ yrs.)*	−0.39 (0.21)	−0.28	−0.38 (0.21)	−0.27	−0.37 (0.21)	−0.27	−0.37 (0.21)	−0.27
**Race**: *White*	0.02 (0.11)	0.03	0.01 (0.11)	0.02	0.01 (0.11)	0.02	0 (0.11)	0
**Poverty**: *<$15K household income*	−0.27 (0.18)	−0.2	−0.22 (0.18)	−0.16	−0.19 (0.18)	−0.14	−0.14 (0.18)	−0.1
**Employment**: *employed*	−0.04 (0.11)	−0.04	−0.02 (0.11)	−0.02	−0.01 (0.11)	−0.01	0.01 (0.11)	0.01
**Education**: *>high school*	−0.02 (0.11)	−0.02	−0.02 (0.11)	−0.02	−0.01 (0.11)	−0.02	−0.01 (0.11)	−0.01
**Smoking**: *smoker*	−0.07 (0.09)	−0.08	−0.08 (0.09)	−0.09	−0.08 (0.09)	−0.1	−0.08 (0.09)	−0.1
**Alcohol**: *risk for chronic drinking*	0.08 (0.17)	0.05	0.07 (0.17)	0.04	0.07 (0.17)	0.04	0.08 (0.17)	0.05
**Obesity**: *obese*	−0.17 (0.11)	−0.17	−0.18 (0.11)	−0.18	−0.18 (0.11)	−0.19	−0.19 (0.11)	−0.2
F (*p*-value)	2.946 (0.003)		2.990 (0.002)		3.070 (0.002)		3.085 (0.002)	
R^2^ (adjusted R^2^)	0.294 (0.194)		0.297(0.197)		0.302 (0.204)		0.303 (0.205)	

** *p* < 0.01.

**Table 4:** Multiple linear regression models to examine the key association between green space, exercise, physical health, and mental health in Illinois.

Dependent Variable	Exercise		Physical Health Problems		Mental Health Problems	
Variables	B (SE)	β	B (SE)	β	B (SE)	β
**Green space**: *time-integrated* *NDVI—place and urban area*	**0.8 (0.28)** **	**0.29**	−0.11 (0.28)	−0.04	−0.12 (0.27)	−0.04
**Urban**: *urban county (yes/no)*	0 (0.02)	0.03	0.03 (0.02)	0.2	0 (0.02)	0.03
**Gender**: *female*	0.16 (0.19)	0.11	0.62 (0.19) ***	**0.41**	0.75 (0.18) ***	**0.47**
**Age**: *elderly (65+ yrs.)*	−0.39 (0.21)	−0.28	−0.38 (0.2)	−0.28	−0.92 (0.2) ***	**−0.62**
**Race**: *White*	0.02 (0.11)	0.03	0 (0.11)	0	0.01 (0.11)	0.01
**Poverty**: *<$15K household income*	−0.27 (0.18)	−0.2	0.38 (0.17) *	**0.28**	0.34 (0.17) *	**0.23**
**Employment**: *employed*	−0.04 (0.11)	−0.04	0.02 (0.11)	0.02	−0.15 (0.1)	−0.16
**Education**: *> high school*	−0.02 (0.11)	−0.02	−0.2 (0.11)	−0.28	−0.06 (0.1)	−0.07
**Smoking**: *smoker*	−0.07 (0.09)	−0.08	0.05 (0.09)	0.06	0.08 (0.08)	0.09
**Alcohol**: *risk for chronic drinking*	0.08 (0.17)	0.05	−0.3 (0.17)	−0.18	0.04 (0.16)	0.03
**Obesity**: *obese*	−0.17 (0.11)	−0.17	0.08 (0.11)	0.09	0.03 (0.1)	0.03
**Exercise**: *any exercise in 30 days*			−0.2 (0.11)	−0.2	0.1 (0.11)	0.09
F (*p*-value)	2.946 (0.003)		3.500 (<0.001)		5.550 (<0.001)	
R^2^ (adjusted R^2^)	0.294 (0.194)		0.353 (0.252)		0.464 (0.380)	

*** *p* < 0.001, ** *p* < 0.01, * *p* <0.05.
